# Effects of Feeding Low Protein Diets with Different Energy-to-Protein Ratios on Performance, Carcass Characteristics, and Nitrogen Excretion of Broilers

**DOI:** 10.3390/ani13091476

**Published:** 2023-04-26

**Authors:** Patrik Strifler, Boglárka Horváth, Nikoletta Such, Valéria Farkas, László Wágner, Károly Dublecz, László Pál

**Affiliations:** 1 Institute of Physiology and Nutrition, Hungarian University of Agriculture and Life Sciences, Georgikon Campus, 8360 Keszthely, Hungary; 2 UBM Feed Company, 2085 Pilisvörösvár, Hungary

**Keywords:** broiler, low protein diet, ideal protein, dietary energy, nitrogen excretion

## Abstract

**Simple Summary:**

Broiler chicken production, providing valuable food products, has also been responsible for ammonia emissions which can lead to environmental pollution worldwide. Besides its economic and animal health advantages, feeding low protein diets can decrease the ammonia emission of broiler production. Even though low-protein diets balanced for amino acids have been used in practice, the ideal energy supply of broilers fed low-protein diets is not known. Therefore, our goal was to determine an ideal dietary energy-to-protein ratio of low-protein diets which does not compromise the production traits and carcass composition of the broilers. Based on our results, the 1.5% crude protein reduction in the diet while maintaining dietary energy constant did not negatively affect the production traits, increased the nitrogen retention, the breast meat yield, and decreased the drip loss of breast meat. Furthermore, the lowest concentration of total-N and uric acid-N in the excreta was obtained when isocaloric diets were fed. Although the low protein diets with reduced energy content led to lower final body weight of broilers, they did not affect the carcass composition, breast meat quality, nitrogen retention, and excreta composition compared to the control diet.

**Abstract:**

This study shows the effects of feeding low protein (LP) diets with different energy-to-protein ratios were evaluated on the production parameters, carcass composition, meat quality, nitrogen retention, and excreta composition of broilers. A total of 576-day-old Ross 308 broilers were fed a control diet (C) and three LP diets containing 1.5% less crude protein than diet C for 41 days. The LP1 treatment was isocaloric with diet C, while the dietary apparent metabolizable energy corrected by nitrogen (AME_n_) levels in the case of the LP2 and LP3 treatments were reduced by 1.5% and 3%, respectively. The LP diets were supplemented with six crystalline essential amino acids (AA) to meet the standardized ileal digestible AA requirements of broilers. The LP1 treatment did not affect the performance parameters of broilers and increased the breast meat yield, the nitrogen retention and decreased drip loss of breast meat and the total-N and uric acid-N nitrogen excretion of birds in comparison with the C group. Although the energy-reduced LP2 and LP3 diets resulted in lower final body weight, they did not affect the carcass composition, breast meat quality, nitrogen retention, and excreta composition of birds compared with the control treatment.

## 1. Introduction

Livestock production has been one of the sources of environmental pollution for land, water, and the atmosphere. Intensive production of poultry meat is also responsible for the emission of ammonia which can lead to water pollution (eutrophication) and soil acidification [1]. The undigested dietary protein and the uric acid from avian nitrogen metabolism are excreted into the environment and may become the source of ammonia emitted from the manure [2]. Therefore, among many other techniques, lowering the crude protein (CP) content of diets can reduce total nitrogen excretions and ammonia emissions of broiler houses. The reduction in dietary CP by 1% can lead to an approximately 10% decrease in N excretion [3]. Besides their lower prices and lower soybean content, broiler diets with reduced CP levels can have many other beneficial effects. A lower intake of nitrogen can lead to a lower water intake of broilers due to the reduced need for water to excrete surplus nitrogen [4]. As a consequence, lower water intake can reduce the risk of wet litter, which in turn can lead to less severe skin dermatitis, causing footpad lesions and breast blisters [5]. Furthermore, the feeding of low protein (LP) diets can decrease the flow of undigested protein into the hindgut, which can result in a lower incidence of dysbiosis and necrotic enteritis [6].

Greater precision in the formulation of diets with LP level is required in order to ensure a balanced amino acid (AA) profile of dietary protein to meet the amino acid need of the broilers and to optimize growth performance and carcass yield. The ‘ideal protein concept’ or ‘ideal amino acid profile’ on a digestible AA basis has been widely used for this purpose [7]. The requirement for limiting essential AAs can be satisfied by the use of crystalline AAs. It is well established that methionine (Met), lysine (Lys), and threonine (Thr) are the three first limiting AA in maize-soybean-based diets [8]. Besides these three essential AAs, valine (Val), isoleucine (Ile), and arginine (Arg) and the first-limiting non-essential amino acids, glycine (Gly) and serine (Ser), should also be considered in LP diets at a certain level of protein reduction [9,10,11]. In addition to the proper balance of dietary essential and non-essential amino acids, phase feeding and pelleting of diets are also inevitable to achieve the goals of intensive production [4]. Diets formulated using these principles allow the reduction of up to 2–3% dietary CP without compromising broiler performance [12].

In spite of their balanced amino acid supply, feeding LP diets may influence body composition negatively. In agreement with the literature data, diets with reduced protein concentration often increase the deposition of abdominal fat [13,14]. Birds excrete excess nitrogen in the form of uric acid synthesized in the liver. This process is quite energy-demanding, so while maintaining dietary apparent metabolizable energy corrected by nitrogen (AME_n_) of LP diets constant, the surplus energy can increase the abdominal fat [13,14]. Until now, the ideal metabolizable energy:crude protein (AME_n_:CP) ratio of LP diets with a balanced amino acid profile has not yet been clarified. The experimental LP diets containing reduced dietary energy with constant AME_n_:CP ratio did not alter the carcass parameters and abdominal fat content of broilers [15,16]. However, the reduction in dietary CP by 1.2–1.5% [15], or 2.0% [16], and the same relative change in AME_n_ significantly reduced the body weight gain of broilers. Therefore, our aim was to investigate the effects of feeding LP diets when the relative reduction in dietary AME_n_ is lower than the relative reduction in dietary CP, which means a novelty over the present knowledge in broiler nutrition. Furthermore, it is not known how the different AME_n_:CP ratios of LP diets affect the nitrogen retention and excreta composition of broilers. Thus, in our experiment, the effects of LP diets with different AME_n_:CP ratios on the production traits, carcass composition, meat quality, nitrogen retention, and excreta composition of broilers.

## 2. Materials and Methods

### 2.1. Experimental Animals and Treatments

A floor pen trial was carried out at the experimental farm of the Institute of Physiology and Nutrition, Georgikon Campus, Hungarian University of Agriculture and Life Sciences (Keszthely, Hungary). A total of 576 one-day-old male broilers (Ross 308) were purchased from a local hatchery (Gallus Ltd., Devecser, Hungary) and divided randomly into 24-floor pens at a stocking rate of 24 broilers per pen (14 bird/m^2^). Broilers were vaccinated against infectious bronchitis (CEVAC BRON), Newcastle disease (CEVAC VITAPEST), and infectious bursal disease (CEVAC TRANSMUNE) in the hatchery using vaccines produced by Ceva (Ceva Santé Animale, 33500 Libourne, France). Chopped wheat straw was used as litter material. The water and feed were provided ad libitum during the whole trial. Diets were fed in mash (starter) and pellet (grower and finisher) form. The climatic conditions and light program, based on the breeder’s guidelines, were computer-controlled and identical for all pens (Aviagen, Newbridge, United Kingdom). The room temperature was set to 34 °C on day 0 and reduced gradually to 24 °C at 18 days of age. The light intensity was 30 lux in the first week and 10 lux thereafter, with a constant day length of 23 h on days 0–7 and 20 h light and 4 h dark period thereafter.

Four dietary treatments consisting of six replicates with 24 broilers in each were established, and experimental diets were fed in the starter (day 0–10), grower (day 11–24), and finisher (day 25–41) phases. A control diet (C) was formulated in line with the breeder’s recommendations for Ross 308 (Aviagen, Newbridge, United Kingdom). Low protein (LP) diets LP1, LP2, and LP3 contained 1.5% less CP than diet C in each dietary phase. This CP reduction in LP diets meant 6.5, 7.0, and 8.0% relative reductions compared to the control diets in the starter, grower, and finisher phases, respectively. The LP1 diet was isocaloric with the control, but the diets LP2 and LP3 had 1.5% and 3.0% lower AME_n_ content. The composition of experimental diets is shown in Table 1. The calculated and measured nutrient content of the diets can be seen in Table 2. The reduction in CP content in LP diets was achieved by reducing the ratio of extracted soybean meal, while the reduced dietary AME_n_ content was ensured by reducing the proportion of sunflower oil. Diets were formulated on the basis of standardized ileal digestible (SID) amino acids in accordance with the ideal protein concept. LP diets were supplemented with six feed-grade crystalline essential amino acids (Lys, Met, Val, Thr, Arg, and Ile) to meet the calculated concentrations of SID AA in the C diets [17]. All diets contained phytase and xylanase enzymes, but no amino acid-releasing impact of these enzymes was considered in the diet formulations.

### 2.2. Measurements

Individual body weight (BW) and feed intake (FI) of broilers per pen were recorded at the end of each dietary phase. Body weight gain (BWG) and feed conversion ratio (FCR) were calculated on a pen basis at the end of each phase as well as for the whole trial period. Mortality and the weight of dead broilers were registered daily during the whole trial.

At day 35, the individual BW of broilers was measured, and two broilers with average body weight from each pen (with individual BW within the range of mean BW per pen ± 2%; 12 broilers per treatment) were selected and transferred to balance cages, where broilers consumed the same finisher diets, but supplemented with 0.5% titanium dioxide as an indigestible internal marker. After four days adaptation period, representative excreta samples were collected from each bird daily for two consecutive days (days 40 and 41). The samples of 12 broilers per treatment were pooled, mixed thoroughly, frozen, and stored at −20 °C until analysis. Before the analyses, excreta was homogenized properly, then the dry matter content, total-N, ammonium-N (NH_4_^+^-N), and uric acid-N contents determined. The dry matter content of excreta samples was measured in a drying oven at 100 °C for 24 h. The total N of excreta was determined according to the Kjeldahl method with Foss-Kjeltec 8400 Analyzer Unit (Nils Foss Allé 1, DK-3400 Hilleroed, Denmark), the ammonium-N by the method of Peters [18], the uric acid-N as described by Marquardt [19]. All N parameters were adjusted to dry matter basis. The sum of ammonium-N and uric acid-N was considered as urinary N content [20]. Feed samples were analyzed for dry matter (ISO 6496), crude protein (ISO 5983-1:2005), phosphorus (ISO 6491), calcium (ISO 6896) content, and amino acid composition (ISO 13903:2005) using the methods of International Organization for Standardization (ISO). The TiO_2_ concentration of experimental diets and excreta samples was determined using a UV-spectroscopy assay [21]. Nitrogen retention was calculated using the following equation [22]: Apparent nitrogen retention = 1 − [([TiO_2_] diet/[TiO_2_] excreta) × ([nitrogen] excreta/[nitrogen] diet)].

At the end of the experiment, two broilers per pen (12 broilers per treatment) representing the average body weight of the pen were selected and slaughtered by cervical dislocation. After evisceration, carcass composition (% of carcass weight, % of breast meat, % of thigh weight, % of abdominal fat) and breast meat quality were determined. The pH of the breast muscle, *Pectoralis major* (*P. major*), was measured immediately after slaughtering (pH_0h_) and after 24 h storage at 4 °C (pH_u_) with a portable pH meter (Testo 205; Testo Ltd., Budapest, Hungary) by inserting a glass electrode directly into the thickest of the breast muscle, always 2 cm from the caudal end of *P. major*. The water-holding capacity of meat was estimated by measuring drip loss of the raw meat: the *P. major* muscle was weighed immediately after slaughter and placed in a plastic bag, hung from a hook, and stored at 4 °C for 24 h. After hanging, the sample was wiped with absorbent paper and weight again. The difference in weight corresponded to the drip loss and was expressed as the percentage of the initial muscle weight [23].

### 2.3. Statistical Analysis

Data of individual broilers were statistically evaluated except in the case of BWG, FI, and FCR, which parameters were evaluated on the basis of pens as experimental units. The averages of examined parameters were analyzed as a completely randomized design by one-way analysis of variance (ANOVA) with dietary treatments as main effects after testing of normal distribution (Kolmogorov–Smirnov test) of data and homogeneity of variances (Levene-test). When the F-test revealed a significant treatment effect, the significant differences between groups were tested by the Tukey HSD test. Regression analysis was used to evaluate the relationship between the calculated dietary starch: CP ratio and the mean FCR of broilers per pen in the starter phase. All statistical analyses were carried out by the software package SPSS 22.0 for Windows (IBM Corp., Armonk, NY, USA). Statistical significance has been declared at *p* < 0.05.

## 3. Results

### 3.1. Performance Parameters

**Figure 1:**
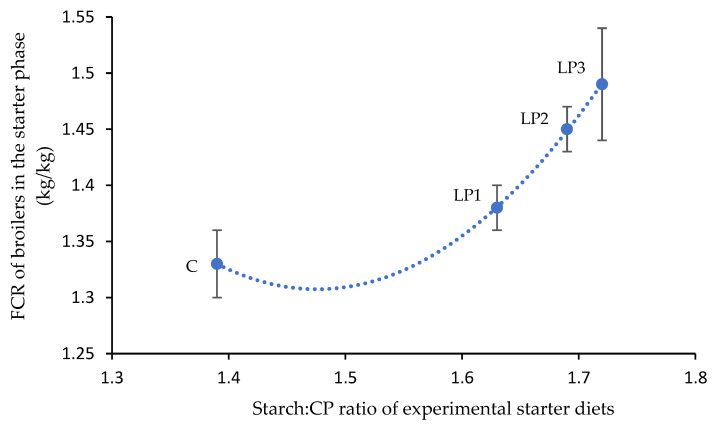
Quadratic relationship (R^2^ = 0.9998; *p* = 0.014) between calculated dietary starch:CP ratio and the mean FCR of animals in the starter phase (mean ± SE; *n* = 6 per treatment) where y = 3.074x^2^ − 9.071x + 8.001. C—control diet; LP1—reduced crude protein levels (−1.5%) and isocaloric AME_n_ content compared to the control diet; LP2—reduced crude protein (−1.5%) and AME_n_ levels (−1.5%) compared to the control diet; LP3—reduced crude protein (−1.5%) and AME_n_ levels (−3%) compared to the control diet.

The performance parameters obtained in the trial are shown in Table 3. The dietary treatments had a significant effect on the BW of broilers at the end of the starter (day 10) and finisher phases (day 41) (*p* < 0.05), while no significant differences were observed between the BW of broilers of different treatment groups at the end of the grower phase. The broilers of treatments C and LP1 achieved a significantly higher BW than the broilers of treatment LP3 after feeding the starter diets (*p* < 0.05). At the end of the finisher phase, the reduction in dietary crude protein without changes of AME_n_ (LP1) did not affect the BW of broilers compared to the C diet. However, the reduction in dietary crude protein together with reduced energy content (LP2 and LP3) led to significantly lower BW of broilers (*p* < 0.05) in relation to the C treatment. Among LP diets, the reduction in dietary AME_n_ had a negative effect on the BW, and a 3% reduction (treatment LP3) decreased the BW of broilers significantly compared to the LP1 group (*p* < 0.05). The values of BWG of broilers in the starter and grower phases were not significantly affected by the dietary treatments. However, the BWG of broilers for the finisher phase and for the whole fattening period was significantly influenced by the experimental treatments (*p* < 0.05). Similar to the BW results, the isocaloric crude protein reduction (LP1) did not result in different BWG of broilers compared to the C treatment. Examining both periods, the broilers of the C group achieved a significantly higher BWG than the broilers of treatments with both crude protein and AME_n_ reduction (LP2 and LP3). The BWG of broilers in the LP treatment groups did not differ significantly. In contrast to BW and BWG data, no significant differences were found in the FI of broilers among the treatments in any phases of the experiment. The dietary treatments affected FCR only in the starter phase when a significantly lower FCR value was observed in the control group than in the treatment LP3 (*p* < 0.05). The increasing starch:CP ratio negatively affected the mean FCR value in the C, LP1, LP2, and LP3 groups, respectively. A quadratic relationship (R^2^ = 0.9998; *p* = 0.014; y = 3.074x^2^ − 9.071x + 8.001) was observed between the calculated dietary starch:CP ratio and the mean FCR of broilers in the starter phase (Figure 1). The increase in the starch:CP ratio between the C and LP1 groups resulted in an increase in the FCR with 0.05. This effect was more pronounced in the LP diets, where the further increase in starch and CP ratio led to an increase in the FCR value of 0.16.

### 3.2. Carcass Characteristics and Breast Meat Quality

The feeding of different experimental diets did not affect the relative carcass weight, the relative thigh weight, and the relative abdominal fat pad ratio (Table 4). The relative breast meat yield of broilers fed the isocaloric and reduced crude protein diet (LP1) was significantly higher than in the C group.

The breast meat quality parameters are shown in Table 5. The experimental treatments did not result in a significant difference in the pH of the breast meat fillet measured either after slaughter or after 24 h (*p* > 0.05). The 24-h drip loss of the breast meat of broilers receiving the LP1 and LP2 diets was significantly lower than in the case of treatment C.

### 3.3. Nitrogen Retention and Excreta Composition

**Figure 2:**
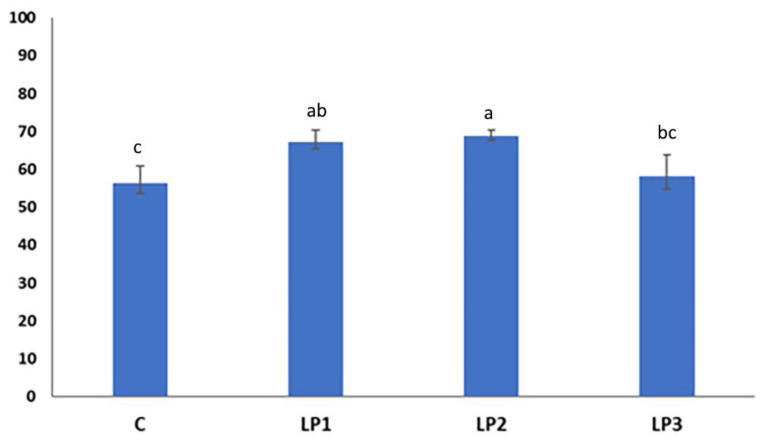
Effect of dietary treatments on N retention of broilers (%; mean ± SE; *n* = 12 broilers per treatment). C—control diet; LP1—reduced crude protein levels (−1.5%) and isocaloric AME_n_ content compared to the control diet; LP2–reduced crude protein (−1.5%) and AME_n_ levels (−1.5%) compared to the control diet; LP3—reduced crude protein (−1.5%) and AME_n_ levels (−3%) compared to the control diet; ^a,b,c^ means with different superscripts are significantly different (*p* < 0.05).

The broilers of treatment LP2 achieved the highest N retention, which proved to be significantly higher than the values in the dietary groups C and LP3 (*p* < 0.05; Figure 2). Furthermore, treatment LP1 also resulted in significantly higher N retention than the control treatment. No significant difference was observed between the N retention values of broilers receiving treatments C and LP3 (*p* > 0.05).

The mean dry matter contents of excreta were 20.9, 20.1, 19.7, and 20.9% in the C, LP1, LP2, and LP3 groups, respectively, and did not show significant differences among treatment groups. The dietary treatments significantly affected the concentration of the fecal-N, uric acid-N, and total-N contents of the excreta (Table 6). Reduction in dietary CP decreased the concentration of these three excreta parameters significantly in comparison to treatments LP1 and C. The decrease in dietary AME_n_ in LP diets increased the concentration of these parameters. In comparison to the LP1 diet, this increase was not significant in the case of the LP2 diet. However, the results of the LP3 group were significantly higher than those of the LP1 group (*p* < 0.05). No significant differences have been found between the results of treatment LP3 and the control treatment. In the case of the NH_4_^+^-N and the urinary-N content of excreta, there was no significant difference between the treatments (*p* > 0.05).

**Figure 3:**
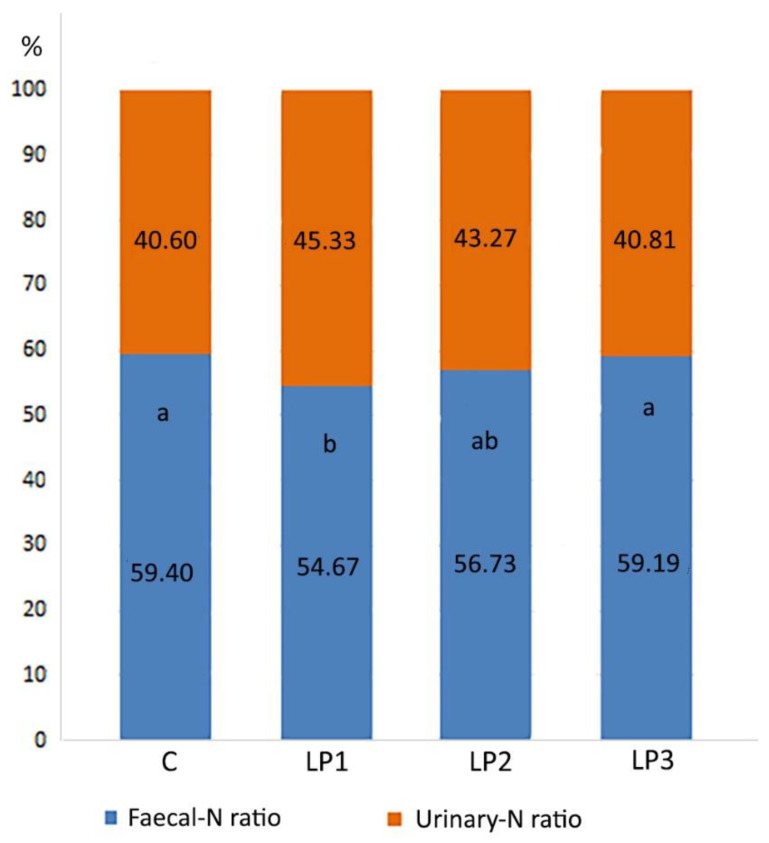
Effects of dietary treatments on the ratio of the fecal- and urinary N content of excreta (%; means). C—control diet; LP1—reduced crude protein levels (−1.5%) and isocaloric AME_n_ content compared to the control diet; LP2—reduced crude protein (−1.5%) and AME_n_ levels (−1.5%) compared to the control diet; LP3—reduced crude protein (−1.5%) and AME_n_ levels (−3%) compared to the control diet; ^a,b^ means of the same treatment with different superscripts are significantly different (*p* < 0.05; Urinary-N:Fecal-N ratio).

The ratio of urinary-N and fecal-N content in excreta followed a similar pattern as in the case of the previously described N forms (Figure 3). Treatment LP1 showed a significantly lower fecal-N ratio compared to treatments C and LP3 (*p* < 0.05). In the case of treatment LP2, no further significant changes were observed.

## 4. Discussion

There are several research results available on the potential of LP broiler diets without change in dietary AME_n_ content (isocaloric diets). However, these results are not consistent due to the differences in the number of experimental phases, feed form, the magnitude of protein reduction, and amino acid composition of the experimental diets. This study shows the feeding of isocaloric control (C) and LP (LP1) diets did not result in significantly different performance traits. Some studies with different limiting factors reported that dietary protein reduction while maintaining dietary energy content constant, impaired the performance of broilers [24,25,26,27,28]. The reasons for the contradictory results are the differences in the trial parameters (age of broilers, the range of CP reduction, amino acids used, etc.). It seems that the rapidly changing requirements of modern hybrids during fattening do not allow the proper adjustment of nutrient supply with only two dietary phases [27] or using mash feed during the whole fattening [28]. Reduction in dietary crude protein by 2% could be achieved without impaired production traits in most trials when at least three-phase feedings were used, pelleted diets were fed, and the diets were balanced in all limiting essential amino acids [26,29,30]. In our case, the essential amino acid contents of diets were set according to the ideal protein concept, and the formulation was based on both total and SID amino acid requirements. Regarding the crystalline amino acid supplementation, not only the four first limiting amino acids (Lys, Met, Thr, and Val) but also L-Arg and L-Ile were used. The general low performance of broilers fed LP diets in some studies can be explained by the fact that crystalline L-Val, L-Arg, and L-Ile were not used [27,31].

In the present experiment, the relative reductions of the dietary AME_n_ in LP2−3 diets were lower than the relative CP reductions of 6.5, 7.0, and 8.0% in the starter, grower, and finisher phases, respectively. This range of dietary energy in LP2 (1.5%) and LP3 (3.0%) diets adversely affected the growth performance and significantly decreased BW at day 10 and day 41 and BWG for the whole experiment, compared to the C group. The AME_n_:CP ratio in LP diets was higher than that in diet C. The broilers fed LP2 and LP3 diets could not compensate for the lower dietary energy by increased feed intake, and therefore, the FCR in these two groups was also depressed in the starter phase. Previous studies investigating the energy supply of broilers fed LP diets used a parallel change of dietary protein and energy while maintaining a constant dietary AME_n_:CP ratio. Unfortunately, this parallel change of both CP and AME_n_ does not allow the separate evaluation of protein and energy effects. Furthermore, these studies had a diverse methodology concerning the essential AA supply of broilers. The feeding isocaloric starter diets with 3% CP reduction (23.4 vs. 20.0%) for three weeks (day 0–21) did not affect the BW and FI but increased the FCR of females of four commercial broiler breeds significantly [32]. These experimental diets with different CP levels had the same total Lys and Met concentrations, but the level of other or digestible AAs was not considered. In a study using six dietary CP regimens with a constant AME_n_:CP ratio, the cumulative growth performance of Ross 308 hybrids significantly decreased when dietary CP was lowered by 1.2–1.5% (relative CP and ME reduction in 7% in each phase). The diets of that trial were not balanced in digestible AAs, and the total Lys and Met + Cys concentrations changed parallel with the dietary CP [15]. In another trial, when Hubbard broiler chickens were fed isocaloric LP diets (2–3% lower CP content, relative reductions of 8–15%; the same digestible Ly, Met, Cys, Thr concentration), lower BWG and impaired FCR was found [16].

Besides the balance of AA and AME_n_:CP ratio, the digestive dynamics of main nutrients, especially starch, lipid, and protein, should be considered in the further development of LP diets. The energy content of broiler diets originates mainly from starch, and the concentration of this nutrient typically increases when dietary CP is reduced [12]. In contrast, dietary lipid level usually decreases together with the protein level. This was the case in this experiment as well. The reduction in dietary AME_n_ from diet LP1 to LP3 was achieved by lowering the lipid content of diets while the starch concentration increased. Hence, the AME_n_:CP ratio decreased; meanwhile, the starch:CP ratio increased with decreasing AME_n_ content in LP1-3 diets. The higher starch:CP ratio can deteriorate the FCR value, as was observed in the starter phase of our experiment in a quadratic manner. A similar quadratic relationship was observed between these two factors from 7 to 35 days in two studies [33,34]. The digestive dynamics of dietary starch and its glucose content has been shown to compete with AA absorption, which may affect the availability of AA for tissue protein accretion [35,36]. Therefore, broiler diets with narrower starch: CP ratios could be beneficial. Lipids, as another important energy source of broiler diets, can influence pellet quality, gastric emptying, and feed intake [37,38,39]. The increasing dietary starch:lipid ratio negatively influenced the feed efficiency of broiler chickens from 21 to 35 days of age [40]. The BWG of broilers to different CP concentrations was modified by dietary lipid levels, but the response was diverse [41]. The optimal lipid and starch ratio of LP diets needs further consideration.

Our results on carcass composition are in line with similar previous studies showing no effect of AA-supplemented LP diets up to 2% CP reduction on the yield of carcass and valuable carcass parts [4,26,42]. The carcass yield of male broilers did not change; however, the relative weight ratios of wings and breast meat were reduced while the leg yield was increased after the feeding of LP diets with a 3% CP reduction [26]. A dietary CP reduction by 2.5% with a balanced AA profile led to a significantly lower carcass yield of females but not of males showing that females may respond more sensitively to marginal AA deficiencies than males [4]. In contrast to the former breast meat yield results, the protein-reduced isocaloric treatment in this experiment significantly increased the relative breast meat yield compared to the control treatment. In the case of LP treatments, the supply of essential amino acids to the pectoral muscle tissue could be more favorable. Liu et al. [12] showed that reduced CP feeding generally increases the AA digestibility in the distal jejunum. This is probably due to the larger ratio of essential crystalline AAs found in the reduced protein diets. The absorption of essential crystalline AAs has been proven to be faster than that of the natural feed AAs, so it can be assumed that a higher plasma essential AA level was available for pectoral muscle synthesis [43]. In the present experiment, probably only the isocaloric LP1 diet provided sufficient energy for the higher incorporation of amino acids into the breast muscle. The energy reductions of LP diets with 1.5 and 3.0% in the present experiment did not influence the carcass, breast meat, and thigh yield significantly. Higher and parallel reductions of AME_n_ (7–15%) and CP also did not alter the carcass yield and yields of carcass parts of broilers fed LP diets with diverse AA supply [15,16]. These results suggest that growth performance parameters are more sensitive to dietary AMEn than the carcass traits if LP diets are fed.

In contrast to many previous trials [13,14,29,44], feeding the isoenergetic LP1 diet did not increase the abdominal fat pad significantly compared to the control. The relative weight of abdominal fat in relation to BW is generally between 2–3% at the end of the broiler fattening. The observed 0.4–0.7% abdominal fat ratio in our experiment is unusually low, and the explanation for this is not clear. The AME_n_ reduction, together with lowering the dietary CP (LP2-3 diets), also did not result in significant changes in this parameter, maybe because of its already very low ratio in the C and LP1 groups. In other studies, the reduction in the dietary energy and CP while maintaining the same energy:CP ratio successfully prevented the accumulation of abdominal fat, but the growth performance of broilers was suppressed [15,16]. It seems that LP diets with reduced AME_n_ content are able to hinder the increase in the abdominal fat ratio, but the appropriate level, using optionally different dietary starch and lipid concentrations, needs further investigation.

The drip loss is one of the parameters characterizing the water-holding capacity of meat which affects its sensory and technological quality. The lower drip loss of breast meat in the LP1 and LP2 groups compared to the C group means lower cooking loss and lower susceptibility to lipid oxidation [45,46]. The negative relationship between drip loss and ultimate pH in poultry meat is well known [46,47]. However, the ultimate pH of breast meat was not significantly different among dietary treatment groups in this study. The higher drip loss of the meat proved to be more acidic with a higher level of glucose, glycogen, and glycolytic potential [46,47,48]. The post-mortem breakdown of the glycogen accumulated in the muscle tissues is responsible for the proper acidity of the meat after slaughter. If an adequate amount of glycogen is not available, the pH of the meat becomes less acidic, and the water-holding capacity of meat is higher. Belloir et al. [49] investigated the effect of AA supply on the meat quality of broiler chickens. According to their results, the drip loss of meat could be associated with excess AAs. After the deamination of the not utilized AAs, the carbon chain is used by the muscle tissue for the synthesis of various carbohydrates, such as glycogen. The lower excess of AAs could contribute to the lower drip loss values in the LP1 and LP2 groups in the present experiment as well.

Based on our knowledge, prior to our experiment, no study had been conducted that investigated the effects of different AME_n_ levels of LP diets on the N retention of broilers and the N composition of the broiler excreta. The N retention values measured in our experiment are in line with the previously published results, which show that the N retention of broiler chickens can be improved by 2–13% using LP diets with reduced CP levels [50,51]. This improvement was observed in broilers fed the LP1 and LP2 diets compared with the control. The LP3 diet with 3% lower energy content did not increase the N retention of broilers, which means that energy was a limiting factor in protein synthesis. The reduction in dietary CP with 1.5% without energy decrease resulted in a 23% decrease in the total-N 26% decrease in uric acid-N excretion. This result shows the impact of LP diets on ammonia emission because the majority of ammonia released from poultry manure originates from the breakdown of uric acid [52]. Similar to our present experiment, Such et al. [30] observed in their research with broiler chickens that besides the total N, the proportion of the urinary N excretion can also be reduced if LP diets are fed. The reduction in AME_n_ in LP diets from LP1 to LP3 increased the total N and uric acid-N concentration of the excreta to the level measured in the C group.

## 5. Conclusions

Based on our results, the 1.5% CP reduction in the starter, grower, and finisher control diets while maintaining dietary AME_n_ constant (LP1) did not negatively affect the production traits and improved the nitrogen retention, the breast meat yield, and the drip loss of breast meat. Furthermore, feeding the isocaloric LP1 diets resulted in the lowest concentration of total-N and uric acid-N excretion of broilers, which is important from an ammonia emission point of view. Although the LP diets with reduced AME_n_ content (LP2 and LP3) led to lower final body weight of broilers, they did not deteriorate the carcass composition, breast meat quality, nitrogen retention, and excretion of broilers compared to the control.

## Figures and Tables

**Table 1:** Composition of experimental diets (%).

	Starter (Day 0–10)				Grower (Day 11–24)				Finisher (Day 25–41)			
Ingredients	C	LP1	LP2	LP3	C	LP1	LP2	LP3	C	LP1	LP2	LP3
Maize	40.83	47.07	48.07	49.08	45.99	52.00	53.02	54.03	51.80	57.66	58.73	59.73
Wheat	10.00	10.00	10.00	10.00	10.00	10.00	10.00	10.00	10.00	10.00	10.00	10.00
Soybean meal ^1^	40.70	35.20	35.10	35.00	35.90	30.70	30.50	30.40	30.70	25.60	25.90	25.80
Sunflower oil	3.60	2.20	1.30	0.40	4.10	2.70	1.80	0.90	3.90	2.60	1.30	0.40
Limestone	1.65	1.68	1.68	1.68	1.39	1.42	1.42	1.42	1.35	1.38	1.38	1.38
Monocalcium phosphate	1.32	1.33	1.33	1.33	1.07	1.08	1.08	1.08	0.93	0.94	0.93	0.93
L-Lysine (Biolys)	0.37	0.63	0.63	0.63	0.24	0.45	0.49	0.49	0.19	0.40	0.38	0.38
DL-Methionine	0.42	0.46	0.46	0.45	0.32	0.36	0.34	0.34	0.20	0.24	0.24	0.23
L-Valine	0.05	0.14	0.14	0.14	0.00	0.06	0.07	0.07	0.00	0.05	0.04	0.04
L-Threonine	0.12	0.19	0.19	0.19	0.06	0.12	0.13	0.13	0.04	0.09	0.09	0.09
L-Arginine	0.00	0.10	0.09	0.09	0.00	0.09	0.11	0.11	0.00	0.09	0.08	0.08
L-Isoleucine	0.00	0.06	0.06	0.06	0.00	0.09	0.09	0.09	0.00	0.06	0.05	0.05
Salt	0.24	0.25	0.25	0.25	0.25	0.25	0.25	0.25	0.25	0.25	0.25	0.25
Sodium bicarbonate	0.10	0.10	0.10	0.10	0.10	0.10	0.10	0.10	0.10	0.10	0.10	0.10
Premix ^2^	0.50	0.50	0.50	0.50	0.50	0.50	0.50	0.50	0.50	0.50	0.50	0.50
Phytase ^3^	0.01	0.01	0.01	0.01	0.01	0.01	0.01	0.01	0.01	0.01	0.01	0.01
NSP enzyme ^4^	0.02	0.02	0.02	0.02	0.02	0.02	0.02	0.02	0.02	0.02	0.02	0.02
Coccidiostat ^5^	0.06	0.06	0.06	0.06	0.06	0.06	0.06	0.06	0.00	0.00	0.00	0.00

C—control diet; LP1—reduced crude protein levels (−1.5%) and isocaloric AME_n_ content compared to the control diet; LP2—reduced crude protein (−1.5%) and AME_n_ levels (−1.5%) compared to the control diet; LP3—reduced crude protein (−1.5%) and AME_n_ levels (−3%) compared to the control diet; ^1^ Soybean meal—extracted soybean meal; ^2^ Premix was supplied by UBM Feed Company (Pilisvörösvár, Hungary). The active ingredients contained in the premix were as follows (per kg of diet): Starter and grower premixes—retinyl acetate—5.0 mg, cholecalciferol—130 g, dl-alpha-tocopherol-acetate—91 mg, menadione—2.2 mg, thiamine—4.5 mg, riboflavin—10.5 mg, pyridoxin HCl—7.5 mg, cyanocobalamin—80 g, niacin—41.5 mg, pantothenic acid—15 mg, folic acid—1.3 mg, biotin—150 g, betaine—670 mg, monensin-Na—110 mg (only grower), narasin—50 mg (only starter), nicarbazin—50 mg (only starter), antioxidant—25 mg, Zn (as ZnSO_4_H_2_O)—125 mg, Cu (as CuSO_4_5H_2_O)—20 mg, Fe (as FeSO_4_H_2_O)—75 mg, Mn (as MnO)—125 mg, I (as KI)—1.35 mg, Se (as Na_2_SeO_3_)—270 g; Finisher premix—retinyl acetate—3.4 mg, cholecalciferol—97 g, dl-alpha-tocopherol-acetate—45.5 mg, menadione—2.7 mg, thiamin—1.9 mg, riboflavin—5.0 mg, pyridoxin HCl—3.2 mg, cyanocobalamin—19 g, niacin—28.5 mg, pantothenic acid—10 mg, folic acid—1.3 mg, biotin—140 g, L-ascorbic acid—40 mg, betaine—193 mg, antioxidant—25 mg, Zn (as ZnSO_4_H_2_O)—96 mg, Cu—9.6 mg, Fe (as FeSO_4_H_2_O)—29 mg, Mn (as MnO)—29 mg, I (as KI)—1.2 mg, Se (as Na_2_SeO_3_)—350 g; ^3^ Axtra^®^ Phy 5000 TPT phytase 500 FTU (Danisco Animal Nutrition & Health, Cedar Rapids, IA 52404, USA); ^4^ Danisco Xylanase 8000 G (Danisco Animal Nutrition & Health, USA); ^5^ Maxiban^®^ G160 premix (Elanco Animal Health, Greenfield, IN 46140, USA).

**Table 2:** Calculated and measured the nutrient content of the experimental diets.

Calculated Nutrients		Starter (Day 0–10)				Grower (Day 11–24)				Finisher (Day 25–41)			
		C	LP1	LP2	LP3	C	LP1	LP2	LP3	C	LP1	LP2	LP3
Crude protein	%	23.00	21.50	21.50	21.50	21.00	19.50	19.50	19.50	19.00	17.50	17.50	17.50
AME_n_	MJ/kg	12.65	12.66	12.46	12.26	13.11	13.09	12.90	12.70	13.40	13.39	13.19	12.99
AME_n_	kcal/kg	3021.4	3023.8	2976.0	2928.3	3131.3	3126.5	3081.1	3033.3	3200.5	3198.1	3150.4	3102.6
Starch	%	32.07	35.08	36.42	36.94	34.82	38.43	39.05	39.67	38.25	41.78	42.44	43.05
Crude fat	%	5.74	4.48	3.63	2.78	6.33	5.08	4.22	3.37	6.25	5.09	3.85	3.00
SID Lysine	%	1.30	1.32	1.32	1.32	1.12	1.12	1.14	1.14	0.98	0.98	0.98	0.98
SID Methionine	%	0.58	0.57	0.57	0.57	0.51	0.51	0.52	0.52	0.47	0.47	0.47	0.47
SID Met + Cys	%	0.94	0.94	0.94	0.94	0.84	0.84	0.84	0.84	0.70	0.70	0.70	0.71
SID Arginine	%	1.39	1.34	1.34	1.34	1.26	1.18	1.20	1.20	1.13	1.08	1.08	1.08
SID Threonine	%	0.83	0.83	0.83	0.83	0.70	0.71	0.72	0.72	0.63	0.62	0.63	0.63
SID Valine	%	0.97	0.98	0.98	0.98	0.85	0.83	0.84	0.84	0.77	0.77	0.77	0.77
SID Isoleucine	%	0.84	0.82	0.82	0.82	0.77	0.72	0.72	0.72	0.69	0.68	0.67	0.67
Ca	%	1.05	1.05	1.05	1.05	0.90	0.90	0.90	0.90	0.85	0.85	0.85	0.85
Pav ^1^	%	0.50	0.50	0.50	0.50	0.45	0.45	0.45	0.45	0.42	0.42	0.42	0.42
AME_n_:CP ratio ^2^		0.55	0.59	0.58	0.57	0.62	0.67	0.66	0.65	0.71	0.77	0.75	0.74
Starch:CP ratio ^3^		1.39	1.63	1.69	1.72	1.66	1.97	2.00	2.03	2.01	2.39	2.43	2.46
Measured nutrients													
Dry matter	%	90.72	90.46	90.24	90.04	89.74	89.20	90.08	90.17	89.29	89.06	88.95	88.65
Crude protein	%	22.72	21.33	21.19	21.20	20.79	19.32	19.34	19.18	18.94	17.41	17.54	17.37
Lysine	%	1.49	1.43	1.44	1.42	1.18	1.16	1.22	1.26	0.98	0.97	1.00	0.97
Methionine	%	0.62	0.61	0.59	0.60	0.55	0.54	0.57	0.59	0.51	0.50	0.52	0.51
Met + Cys	%	0.98	0.95	0.93	0.94	0.84	0.84	0.88	0.91	0.82	0.78	0.81	0.79
Arginine	%	1.55	1.45	1.39	1.42	1.27	1.27	1.45	1.36	1.29	1.12	1.19	1.13
Threonine	%	0.92	0.90	0.93	0.91	0.77	0.78	0.80	0.82	0.71	0.67	0.66	0.65
Valine	%	1.06	1.10	1.05	1.05	0.90	0.88	0.94	0.95	0.85	0.78	0.80	0.78
Isoleucine	%	0.94	0.96	0.91	0.93	0.76	0.78	0.79	0.81	0.74	0.69	0.70	0.68
Ca	%	1.05	1.03	1.08	1.05	0.91	0.90	0.93	0.90	0.89	0.85	0.86	0.87
P	%	0.70	0.70	0.69	0.70	0.56	0.56	0.60	0.61	0.51	0.48	0.51	0.48

C—control diet; LP1—reduced crude protein levels (−1.5%) and isocaloric AME_n_ content compared to the control diet; LP2—reduced crude protein (−1.5%) and AME_n_ levels (−1.5%) compared to the control diet; LP3—reduced crude protein (−1.5%) and AMEn levels (−3%) compared to the control diet; ^1^ Available P; ^2^ Ratio of dietary AME_n_ and crude protein concentration; ^3^ Ratio of dietary starch and crude protein concentration.

**Table 3:** Performance parameters of birds in the starter, grower, and finisher phase and in the whole experiment (mean ± SE).

	Treatment	Starter (Day 0–10)	Grower (Day 11–24)	Finisher (Day 25–42)	Overall (Day 0–41)
Body weight ^1^ (g)	C	251.6 ± 3.5 ^a^	1275.5 ± 11.6	2928.1 ± 23.5 ^a^	-
	LP1	248.9 ± 3.8 ^a^	1309.4 ± 12.9	2855.7 ± 26.2 ^ab^	-
	LP2	238.9 ± 3.9 ^ab^	1257.3 ± 15.1	2770.1 ± 28.8 ^bc^	-
	LP3	233.9 ± 4.1 ^b^	1272.1 ± 15.3	2744.0 ± 28.4 ^c^	-
	*p*	0.003	NS	<0.001	-
Body weight gain (g)	C	201.9 ± 6.8	1029.6 ± 14.0	1643.6 ± 20.8 ^a^	2875.2 ± 30.8 ^a^
	LP1	199.9 ± 2.9	1031.5 ± 23.2	1576.4 ± 39.0 ^ab^	2807.8 ± 43.3 ^ab^
	LP2	191.9 ± 3.3	1018.4 ± 16.4	1494.7 ± 17.6 ^b^	2705.0 ± 25.6 ^b^
	LP3	186.9 ± 6.3	1028.4 ± 14.7	1470.6 ± 32.1 ^b^	2685.9 ± 41.2 ^b^
	*p*	NS	NS	0.001	0.004
Feed intake (g)	C	268.4 ± 3.5	1296.6 ± 17.7	3109.7 ± 50.1	4674.7 ± 57.0
	LP1	276.2 ± 1.6	1275.5 ± 11.2	3139.4 ± 80.3	4691.1 ± 75.0
	LP2	277.8 ± 3.9	1275.0 ± 19.8	2943.2 ± 57.7	4496.0 ± 60.9
	LP3	276.6 ± 4.4	1267.4 ± 25.6	2923.4 ± 39.8	4467.4 ± 62,4
	*p*	NS	NS	NS	NS
FCR (kg/kg)	C	1.33 ± 0.03 ^b^	1.26 ± 0.01	1.96 ± 0.04	1.66 ± 0.02
	LP1	1.38 ± 0.02 ^ab^	1.24 ± 0.02	2.01 ± 0.03	1.70 ± 0.04
	LP2	1.45 ± 0.02 ^ab^	1.23 ± 0.01	2.02 ± 0.03	1.69 ± 0.01
	LP3	1.49 ± 0.05 ^a^	1.23 ± 0.02	2.05 ± 0.05	1.69 ± 0.03
	*p*	0.014	NS	NS	NS

^1^ Body weight of broilers was measured at the end of the feeding phases, at days 10, 24, and 41. C—control diet; LP1—reduced crude protein levels (−1.5%) and isocaloric AME_n_ content compared to the control diet; LP2—reduced crude protein (−1.5%) and AME_n_ levels (−1.5%) compared to the control diet; LP3—reduced crude protein (−1.5%) and AME_n_ levels (−3%) compared to the control diet; ^a,b,c^ means with different superscripts of the same column are significantly different (*p* < 0.05); NS—nonsignificant (*p* > 0.05).

**Table 4:** Carcass weight and composition ^1^ (mean ± SE; *n* = 12 broilers per treatment).

Treatment	Carcass Weight (%)	Breast Meat Yield (%)	Thigh Weight (%)	Abdominal Fat (%)
C	65.41 ± 0.31	21.41 ± 0.31 ^b^	19.63 ± 0.22	0.49 ± 0.08
LP1	66.85 ± 0.41	23.20 ± 0.49 ^a^	19.41 ± 0.38	0.70 ± 0.10
LP2	66.08 ± 0.26	22.65 ± 0.26 ^ab^	19.22 ± 0.23	0.49 ± 0.07
LP3	65.94 ± 0.46	22.78 ± 0.54 ^ab^	19.27 ± 0.35	0.62 ± 0.06
Significance (*p*)	NS	0.026	NS	NS

C—control diet; LP1—reduced crude protein levels (−1.5%) and isocaloric AME_n_ content compared to the control diet; LP2—reduced crude protein (−1.5%) and AME_n_ levels (−1.5%) compared to the control diet; LP3—reduced crude protein (−1.5%) and AME_n_ levels (−3%) compared to the control diet; ^1^ values expressed as a percentage of live BW; ^a,b^ means with different superscripts of the same column are significantly different (*p* < 0.05); NS—nonsignificant (*p* > 0.05).

**Table 5:** Breast meat quality parameters (mean ± SE; *n* = 12 broilers per treatment).

Treatment	pH_0h_ ^1^	pH_u_ ^2^	Drip Loss (%)
C	6.55 ± 0.03	5.73 ± 0.03	2.27 ± 0.17 ^a^
LP1	6.61 ± 0.02	5.78 ± 0.03	1.63 ± 0.07 ^b^
LP2	6.59 ± 0.03	5.73 ± 0.04	1.84 ± 0.09 ^b^
LP3	6.56 ± 0.03	5.76 ± 0.03	1.90 ± 0.06 ^ab^
Significance (*p*)	NS	NS	0.002

C—control diet; LP1—reduced crude protein levels (−1.5%) and isocaloric AME_n_ content compared to the control diet; LP2—reduced crude protein (−1.5%) and AME_n_ levels (−1.5%) compared to the control diet; LP3—reduced crude protein (−1.5%) and AME_n_ levels (−3%) compared to the control diet; ^1^ pH_0h_ = pH measured immediately after slaughter; ^2^ pH_u_ = pH measured after 24 h storage at 4 °C; ^a,b^ means with different superscripts of the same column are significantly different (*p* < 0.05); NS—nonsignificant (*p* > 0.05).

**Table 6:** The concentration of N-forms in broiler excreta (mean ± SE; *n* = 12 broilers per treatment).

Treatment	Fecal-N	NH_4_^+^-N	Uric Acid-N	Urinary-N ^1^	Total-N
	mg/g Dry Matter				
C	32.75 ± 2.33 ^a^	4.58 ± 0.26	17.65 ± 1.13 ^a^	22.23 ± 1.34	54.98 ± 3.62 ^a^
LP1	21.96 ± 1.26 ^b^	5.20 ± 0.41	13.07 ± 0.76 ^b^	18.27 ± 1.12	40.24 ± 2.32 ^b^
LP2	25.94 ± 1.84 ^ab^	4.76 ± 0.40	15.03 ± 1.09 ^ab^	19.79 ± 1.44	45.73 ± 3.15 ^ab^
LP3	33.42 ± 2.45 ^a^	4.92 ± 0.55	18.25 ± 1.49 ^a^	23.16 ± 2.01	56.58 ± 4.38 ^a^
Significance (*p*)	<0.001	NS	0.009	NS	0.004

^1^ The sum of NH_4_^+^-N and uric acid-N was considered as urinary-N content; C—control diet; LP1—reduced crude protein levels (−1.5%) and isocaloric AME_n_ content compared to the control diet; LP2—reduced crude protein (−1.5%) and AME_n_ levels (−1.5%) compared to the control diet; LP3—reduced crude protein (−1.5%) and AME_n_ levels (−3%) compared to the control diet; ^a,b^ means with different superscripts of the same column are significantly different (*p* < 0.05); NS—nonsignificant (*p* > 0.05).

## Data Availability

Data supporting the reported results is contained within the article.
